# Influencing factors for pcr-fluorescent probe detection results in patients with lymph node tuberculosis: A retrospective observational study

**DOI:** 10.1097/MD.0000000000048623

**Published:** 2026-05-01

**Authors:** Qian Li, Peijia Luo, Dangze Sun, Chao Ding, Leipeng Ren

**Affiliations:** a Department of Thoracic Surgery, Xi’an Chest Hospital, Xi’an, Shaanxi, China.

**Keywords:** age, extrapulmonary tuberculosis, PCR-fluorescent probes, restriction cubic spline, T lymphocyte subsets

## Abstract

Lymph node tuberculosis (LNTB) is the most common form of extrapulmonary tuberculosis, and its diagnosis and drug resistance screening remain challenging due to low positivity rates of conventional microbiological tests. The polymerase chain reaction (PCR)-fluorescent probe method has been widely used for rapid detection of *Mycobacterium tuberculosis*, but factors influencing its detection positivity in LNTB remain insufficiently explored. This study aimed to investigate the influencing factors of PCR-fluorescent probe detection positivity and preliminarily explore the dose‑response relationships between age, T lymphocyte subsets, and PCR-fluorescent probe results in patients with LNTB. The clinical data of 411 patients with LNTB admitted to Xi’an Chest Hospital from November 30, 2018 to June 17, 2024 were retrospectively analyzed. The influencing factors were screened by univariate + multivariate logistic regression analysis. Furthermore, the dose-response relationship between PCR-fluorescent probe detection results and age and T lymphocyte subsets was analyzed using a restricted cubic spline model. The results of multivariate logistic regression analysis indicated that obtaining samples through surgery (odds ratio [OR] = 3.01, 95% confidence interval [CI] 1.45–6.26), the number of samples (OR = 3.03, 95% CI 1.69–5.44), the second quartile of age (OR = 2.95, 95% CI: 1.41–6.19), and the third quartile of cluster of differentiation (CD)4+ CD8+ T helper/T suppressor cells (OR = 2.75, 95% CI: 1.29–5.85) increased the risk of positive PCR- fluorescent probe results. In contrast, the third quartile of total T lymphocyte CD3+ (OR = 0.42, 95% CI: 0.21–0.87) decreased the risk of positive PCR-fluorescent probe results. Additionally, the second, third, and fourth quartile indices of T helper lymphocytes CD3+ CD4+ all reduced the risk of positive PCR-fluorescent probe results (*P*_trend_ < .05). There was a nonlinear dose-response relationship between age (*P*_overall trend_ < .001, *P*_nonlinear_ < .001), total T lymphocyte CD3+ (*P*_overall trend_ = .017, *P*_nonlinear_ = .014), and the positive risk of PCR-fluorescent probe detection results. Conversely, a linear dose-response relationship was observed between CD3+ CD4+ (*P*_overall trend_ = .026, *P*_nonlinear_ = .116) and the positive risk of PCR-fluorescent probe detection results. The positive outcomes of PCR-fluorescence probe detection are facilitated by obtaining samples through surgery and an increased number of samples. Furthermore, both linear and nonlinear dose-response relationships were observed between age, T lymphocyte subsets, and the results of PCR-fluorescence probe detection.

## 1. Introduction

Tuberculosis (TB) is a chronic infectious disease caused by the invasion of *Mycobacterium tuberculosis* (MTB) into the human body. It primarily affects the lungs but can also spread to other organs, a condition known as extrapulmonary TB. Among these, lymph node tuberculosis (LNTB) is the most common form,^[1]^ and its incidence has shown no downward trend in recent years.^[2,3]^ Diagnosing extrapulmonary TB is more challenging than pulmonary TB, often leading to longer treatment durations and a higher likelihood of relapse. The molecular biology polymerase chain reaction (PCR)-fluorescent probe method has a high positive rate for the detection of MTB nucleic acid.^[4,5]^ Currently, there are differences and room for improvement in the molecular biology test results among different populations of extrapulmonary TB patients. Research focusing on factors affecting PCR-fluorescent probe test results is relatively scarce.^[6]^ Therefore, this study conducts a retrospective analysis using clinical data from LNTB patients. Univariate and multivariate logistic regression were used to screen influencing factors, and restricted cubic spline (RCS) model analysis was employed to explore the dose-response relationship between age, T lymphocyte subset-related indicators, and PCR-fluorescent probe test results. This aims to improve the rate of obtaining positive PCR results and drug resistance testing for LNTB patients, providing a reference for guiding subsequent treatment.

## 2. Materials and methods

### 2.1. Study design and participants

**Figure 1.**
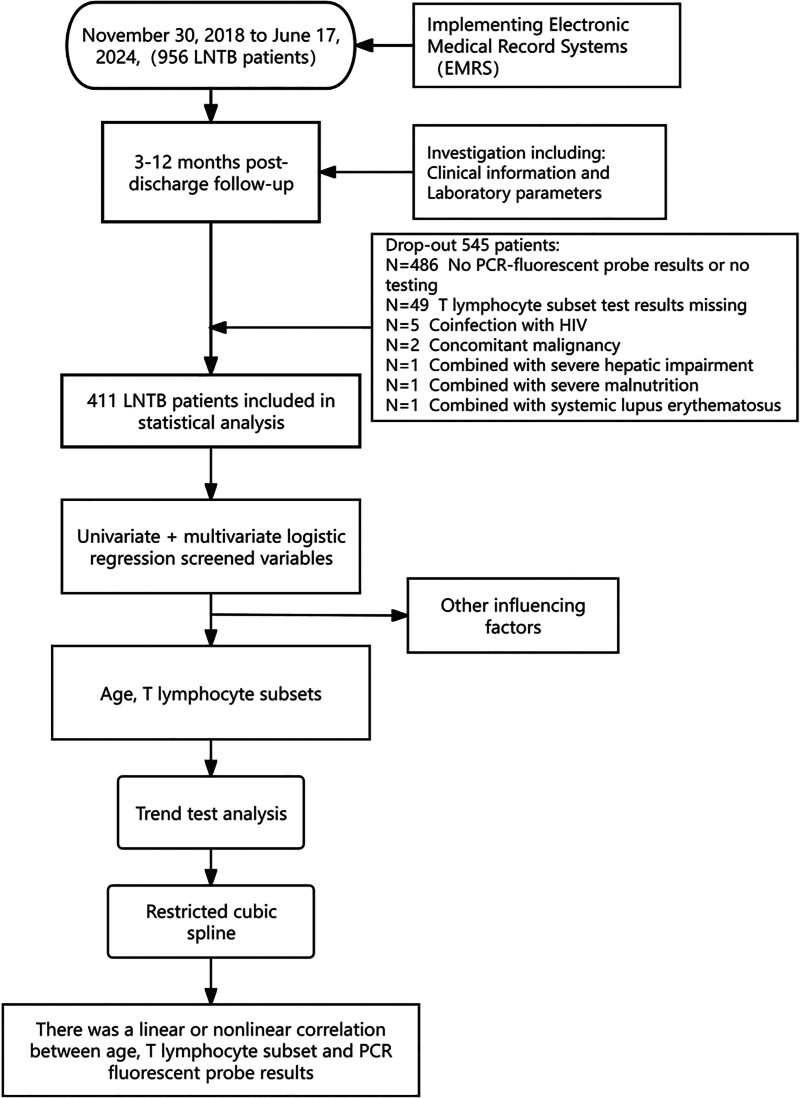
Study flow chart of patient inclusion. EMRS = electronic medical record systems, HIV = human immunodeficiency virus, LNTB = lymph node tuberculosis..

A total of 411 patients with confirmed superficial LNTB treated at Xi’an Chest Hospital from November 30, 2018 to June 17, 2024 were collected as subjects for this study. The diagnostic criteria for superficial lymph node TB are as follows: histopathology showing granulomatous inflammation with or without caseating necrosis; MTB for mycobacterial culture and species identification and/or any molecular biology tests [including GeneXpert MTB/rifampicin (Cepheid, USA), fluorescent PCR (Beijing Boao Crystal Code Biotechnology Co., Ltd.)].^[7,8]^ Inclusion Criteria: patients included in the study must meet diagnostic criteria for LNTB; medical records and information are complete. Exclusion Criteria: PCR-fluorescent probe, T lymphocyte analysis missing results; concurrent malignancy or human immunodeficiency virus. Severe disease of vital organs (e.g., severe liver dysfunction, severe malnutrition, and systemic lupus erythematosus). A flowchart for this study is shown in Figure 1。

### 2.2. Ethical approval

The Declaration of Helsinki was followed in conducting this study. The Ethics Committee of Xi’an Chest Hospital approved the research protocol (S2024-006-01). To protect privacy, no personally identifiable information is provided. Due to the retrospective nature of this study and the fact that all patients were anonymous participants, this study obtained an informed consent waiver from the Ethics Committee of Xi’an Chest Hospital. In addition, during the implementation of this study, this study was carried out in accordance with national legislative and institutional requirements.

### 2.3. Data Collection

Data were extracted from the hospital’s electronic information system. Demographic characteristics were obtained upon admission. Complications were determined through patient self-report or previous diagnosis, and blood samples were collected within 24 hours of admission for laboratory testing. The data include: Demographic characteristics: gender, age, body mass index (BMI), smoking, alcohol consumption; TB-related information: disease duration, antituberculosis treatment time before testing, obtaining samples through surgery, TB of other organs, antituberculosis treatment regimen [standard quadruple therapy with isoniazid, rifampicin, pyrazinamide, ethambutol; nonstandard quadruple therapy], initial treatment/retreatment (Initial treatment refers to one of the following conditions: a patient who has never been treated with antituberculosis drugs for TB; a patient who is undergoing standard chemotherapy and has not completed the course; a patient who has had irregular chemotherapy for less than 1 month. Retreatment refers to one of the following conditions: a patient who has been treated with antituberculosis drugs irrationally or irregularly for more than 1 month due to TB; a patient with initial treatment failure or relapse), drug resistance status (including Isoniazid, Rifampicin, Ethambutol, Streptomycin, Quinolones); Comorbidities: diabetes, hypertension, liver or gallbladder diseases; Laboratory data: anemia (adult males: < 120 g/L, adult females (nonpregnant): < 110 g/L; pregnant women: < 100g/L; children aged 6 to 59 months: <110 g/L, hematocrit < 0.33), hypoproteinemia (total protein < 60.0 g/L, albumin < 25 g/L), adenosine deaminase, T lymphocyte subset analysis (total T lymphocytes cluster of differentiation [CD]3+, T helper lymphocytes CD3+ CD4+, T suppressor lymphocytes CD3+ CD8+, T helper/T suppressor cell ratio CD4+/CD8+); Number of samples: single sample: purulent sample or granulation tissue sample; double samples: granulation tissue + granulation tissue sample or granulation tissue + purulent sample or purulent + purulent sample.

### 2.4. Sample and data processing

PCR-fluorescent probe method and sample processing^[9]^: the instrument used is the ABI7500 Real-Time PCR System (Applied Biosystems, USA), with supporting reagents being *Mycobacterium* nucleic acid detection kits (Beijing Biotech Co., Ltd., batch numbers 0112161708 and 0105171801). Patient samples are processed according to the instructions provided in the kit manual. Tissue samples are homogenized using a tissue grinder followed by resuspension in saline; purulent samples are mixed with an appropriate amount of saline for resuspension.^[10]^ Additionally, 0.5 McFarland units of H37Rv bacterial suspension are extracted for nucleic acid adjustment to 1 × 10^6^ U/mL as a positive control before testing on the machine. BMI Classification: Q1: BMI < 18.5; Q2: 18.5 to < 24.0; Q3: 24.0 to < 28; Q4: ≥ 28. Age and T lymphocyte subset-related indicators are grouped based on quartiles (Q1, Q2, Q3, Q4), represented by the median values of each group’s variables.

### 2.5. Statistical Analysis

Data analysis and processing were conducted using SPSS 27.0 (IBM Corp., Armonk) software and R 4.3.2 (R Foundation for Statistical Computing, Vienna, Austria) software. For normally distributed quantitative data, “mean ± standard deviation” was used for description, and the *t*-test was employed to compare differences between groups; for skewed distribution quantitative data, “median (interquartile range) [M(Q_25_, Q_75_)]” was used for description, and the Mann–Whitney U test was applied to compare differences between groups; categorical variables were described as “number of cases, constituent ratio/percentage (%),” and the Chi-square test was used to compare differences between groups; For some independent variables with < 5% missing data, single imputation is employed. PCR-fluorescent probe results were taken as the dependent variable, and single-factor + multi-factor logistic regression analysis was used to identify independent influencing factors. Covariates with *P* < .05 in univariate logistic regression were included in the final multivariate model. Covariate selection was also determined by clinical relevance and evidence from previous studies. Although multivariate adjustment was conducted, residual confounding cannot be fully excluded, especially for immune-related indicators, disease severity, and prior antituberculosis treatment exposure, which could not be fully captured in this retrospective study. Age and T lymphocyte subset data were grouped using the quartile method, with the median of each group’s variables entered as continuous variables into the regression model for trend testing. RCS models combined with logistic regression analysis were applied to examine the dose-response relationships between age, total T lymphocytes, T helper lymphocytes, T helper/T suppressor lymphocytes, and PCR-fluorescent probe results. A *P* value of < .05 was considered statistically significant.

## 3. Results

### 3.1. Baseline characteristics of the study subjects

A total of 411 study subjects were included, with an average age of 36.31 ± 14.72 years, including 152 males (36.98%) and 259 females (63.02%). The differences in obtaining samples through surgery, concurrent hypertension, number of samples, drug resistance, and total T lymphocyte CD3 + were all statistically significant (*P* < .05; Table 1).

**Table 1:** Baseline characteristics of the study participants by PCR-fluorescent probe results.

Variables	Total (n = 411)	PCR		Statistic	*P*
		Negative (n = 114)	Positive (n = 297)		
Age, Mean ± SD	36.31 ± 14.72	37.75 ± 16.13	35.75 ± 14.13	*t* = 1.16	.248
Gender, n (%)				*χ^2^* = 0.42	.517
Female	152 (36.98)	45 (39.47)	107 (36.03)		
Male	259 (63.02)	69 (60.53)	190 (63.97)		
BMI, Mean ± SD	21.49 ± 3.05	21.82 ± 3.11	21.36 ± 3.02	*t* = 1.37	.172
Course of disease (months), M (Q_25_, Q_75_)	4.00 (1.50, 12.00)	5.00 (2.00, 12.00)	4.00 (1.50, 12.00)	*Z* = −1.26	.208
Antituberculosis treatment time before testing (day), M (Q_25_, Q_75_)	32.00 (16.00, 96.50)	29.50 (15.00, 90.00)	33.00 (17.00, 97.00)	*Z* = −0.87	.382
ADA, M (Q_25_, Q_75_)	10.00 (8.00, 12.00)	10.00 (8.00, 13.00)	10.00 (8.00, 12.00)	*Z* = −0.45	.652
Initial treatment or retreatment, n (%)				*χ*^2^ = 0.08	.783
Initial treatment	201 (48.91)	57 (50.00)	144 (48.48)		
Retreatment	210 (51.09)	57 (50.00)	153 (51.52)		
obtaining samples through surgery, n (%)				*χ*^2^ = 7.60	.006
No	47 (11.44)	21 (18.42)	26 (8.75)		
Yes	364 (88.56)	93 (81.58)	271 (91.25)		
Antituberculosis treatment regimen (H-R-Z-E), n (%)				*χ*^2^ = 0.05	.828
Yes	148 (36.01)	42 (36.84)	106 (35.69)		
No	263 (63.99)	72 (63.16)	191 (64.31)		
Smoking, n (%)				*χ*^2^ = 1.18	.278
No	380 (92.46)	108 (94.74)	272 (91.58)		
Yes	31 (7.54)	6 (5.26)	25 (8.42)		
Drinking, n (%)				*χ*^2^ = 0.00	1.000
No	405 (98.54)	112 (98.25)	293 (98.65)		
Yes	6 (1.46)	2 (1.75)	4 (1.35)		
Anemia, n (%)				*χ*^2^ = 2.52	.112
No	341 (82.97)	100 (87.72)	241 (81.14)		
Yes	70 (17.03)	14 (12.28)	56 (18.86)		
Hypoproteinemia, n (%)				*χ*^2^ = 0.79	.374
No	366 (89.05)	99 (86.84)	267 (89.90)		
Yes	45 (10.95)	15 (13.16)	30 (10.10)		
Tuberculosis of other organs, n (%)				*χ*^2^ = 3.67	.055
No	203 (49.39)	65 (57.02)	138 (46.46)		
Yes	208 (50.61)	49 (42.98)	159 (53.54)		
Diabetes, n (%)				*χ*^2^ = 2.79	.095
No	400 (97.32)	108 (94.74)	292 (98.32)		
Yes	11 (2.68)	6 (5.26)	5 (1.68)		
Hypertension, n (%)				*χ*^2^ = 4.36	.037
No	390 (94.89)	104 (91.23)	286 (96.30)		
Yes	21 (5.11)	10 (8.77)	11 (3.70)		
Hepatobiliary diseases, n (%)				*χ*^2^ = 2.31	.128
No	322 (78.35)	95 (83.33)	227 (76.43)		
Yes	89 (21.65)	19 (16.67)	70 (23.57)		
Number of samples, n (%)				*χ*^2^ = 15.37	**<** .001
Single	279 (67.88)	94 (82.46)	185 (62.29)		
Double	132 (32.12)	20 (17.54)	112 (37.71)		
Drug resistance, n (%)				*χ*^2^ = 17.15	**<** .001
No	353 (85.89)	111 (97.37)	242 (81.48)		
Yes	58 (14.11)	3 (2.63)	55 (18.52)		
Total T lymphocytes CD3+, M (Q_25_, Q_75_)	1101.00 (841.00, 1456.50)	1222.50 (927.50, 1488.50)	1050.00 (808.00, 1439.00)	*Z* = −2.15	.031
T helper CD3 + CD4+, M (Q_25_, Q_75_)	640.00 (468.50, 838.50)	708.50 (535.25, 864.25)	623.00 (454.00, 816.00)	*Z* = −1.87	0.061
T suppressor lymphocyte CD3 + CD8+, M (Q_25_, Q_75_)	413.00 (303.00, 578.50)	436.00 (325.25, 631.25)	401.00 (298.00, 571.00)	*Z* = −1.83	.068
T helper/T suppressor cells, M (Q_25_, Q_75_)	1.55 (1.19, 2.05)	1.47 (1.15, 2.05)	1.59 (1.21, 2.03)	*Z* = −0.85	.395

The normal range of each index: body mass index: 18.5–23.9kg/m^2^, adenosine deaminase: 0–15 μ/L, total T lymphocytes CD3+: 690–2540 cells/ μL, T helper lymphocytes CD3+ CD4+: 410–1590 cells/μL, T inhibitory lymphocyte CD3 + CD8 + 190–1140 cells/μL, T helper/T inhibitory cells: 1.40–2.00 cells/μL.

ADA = adenosine deaminase, BMI = body mass index, CD3+ = total T lymphocytes, CD3+CD4+ = T helper lymphocytes, CD3+CD8+ = T suppressor lymphocytes, H-R-Z-E = isoniazid-rifampicin-pyrazinamide-ethambutol, n = number of participants, PCR = polymerase chain reaction, SD = standard deviation.

### 3.2. Univariate and multivariate logistic regression results of PCR-Fluorescent probe

Taking the PCR-fluorescent probe result as the dependent variable, differences in number of samples, obtaining samples through surgery, age, total T lymphocyte CD3+, T helper lymphocyte CD3 + CD4+, and T helper/T suppressor cell CD4+ CD8+ were all statistically significant in both univariate and multivariate logistic regression (*P* < .05). However, gender, BMI, course of disease, smoking, drinking, anemia, hypoproteinemia, TB of other organs, hypertension, diabetes, hepatobiliary diseases, antituberculosis treatment regimen, T suppressor lymphocyte CD3+ CD8+, and other indicators showed no statistical significance (*P* > .05), as shown in Tables 2 and 3.

**Table 2:** Univariate logistic regression analysis results of PCR-Fluorescent probe.

Variables	*β*	SE	*Waldχ*2	*P*	OR (95% CI)
Gender, n (%)					
Female					1.00 (Reference)
Male	0.15	0.23	0.65	.517	1.16 (0.74–1.81)
Course of disease (months)	−0.01	0.00	−2.40	.016	0.99 (0.99–0.99)
Antituberculosis treatment time before testing (d)	0.00	0.00	0.75	.452	1.00 (1.00–1.00)
Number of samples					
Single					1.00 (Reference)
Double	1.05	0.27	3.82	**<** .001	2.85 (1.66–4.87)
obtaining samples through surgery					
No					1.00 (Reference)
Yes	0.86	0.32	2.70	.007	2.35 (1.26–4.38)
Antituberculosis treatment regimen (H-R-Z-E)					
Yes					1.00 (Reference)
No	0.05	0.23	0.22	.828	1.05 (0.67–1.65)
Initial treatment or retreatment					
Initial treatment					1.00 (Reference)
Retreatment	0.06	0.22	0.28	.783	1.06 (0.69–1.64)
Smoking					
No					1.00 (Reference)
Yes	0.50	0.47	1.07	.283	1.65 (0.66–4.15)
Anemia					
No					1.00 (Reference)
Yes	0.51	0.32	1.58	.115	1.66 (0.88–3.12)
Drinking					
No					1.00 (Reference)
Yes	−0.27	0.87	−0.31	.758	0.76 (0.14–4.23)
Hypoproteinemia					
No					1.00 (Reference)
Yes	−0.30	0.34	−0.89	.376	0.74 (0.38–1.44)
Tuberculosis of other organs					
No					1.00 (Reference)
Yes	0.42	0.22	1.91	.056	1.53 (0.99–2.36)
Hypertension					
No					1.00 (Reference)
Yes	−0.92	0.45	−2.03	.042	0.40 (0.17–0.97)
Diabetes					
No					1.00 (Reference)
Yes	−1.18	0.62	−1.91	.056	0.31 (0.09–1.03)
Hepatobiliary diseases, n (%)					
No					1.00 (Reference)
Yes	0.43	0.29	1.51	.130	1.54 (0.88–2.70)
BMI					
Q2 (< 18, 5)					1.00 (Reference)
Q3 (18.5–23.9)	−0.40	0.28	−1.44	.149	0.67 (0.39–1.16)
Q1 (24.0–27.9)	0.13	0.34	0.38	.702	1.14 (0.59–2.20)
Q4 (> 28.0)	0.08	0.83	0.10	.921	1.09 (0.21–5.50)
Age					
Q1 (22, 4–25)					1.00 (Reference)
Q2 (29, 26–31)	0.86	0.35	2.48	.013	2.37 (1.20–4.67)
Q3 (36, 32–45)	0.62	0.32	1.96	.050	1.86 (1.00–3.45)
Q4 (56, 46–83)	−0.32	0.29	−1.09	.275	0.73 (0.41–1.29)
Total T lymphocytes CD3+					
Q1 (673.5, 238–834)					1.00 (Reference)
Q2 (971, 841–1100)	0.13	0.34	0.38	.706	1.14 (0.58–2.22)
Q3 (1247, 1101–1456)	−0.70	0.31	−2.23	.026	0.50 (0.27–0.92)
Q4 (1743, 1457–4058)	−0.44	0.32	−1.37	.170	0.65 (0.35–1.21)
T helper CD3 + CD4+					
Q1 (371, 112–468)					1.00 (Reference)
Q2 (558.5, 469–637)	−0.56	0.34	−1.68	.093	0.57 (0.29–1.10)
Q3 (722, 640–838)	−0.69	0.33	−2.08	.038	0.50 (0.26–0.96)
Q4 (1015, 839–1799)	−0.78	0.33	−2.36	.018	0.46 (0.24–0.88)
T suppressor lymphocyte CD3 + CD8+					
Q1 (216.5, 87–302)					1.00 (Reference)
Q2 (346.0, 303–412)	0.15	0.33	0.46	.645	1.17 (0.61–2.24)
Q3 (474.0, 413–578)	−0.30	0.31	−0.96	.337	0.74 (0.40–1.37)
Q4 (710.0, 579–2077)	−0.46	0.31	−1.49	.138	0.63 (0.34–1.16)
T helper/ T suppressor cells					
Q1 (0.93, 0.35–1.18)					1.00 (Reference)
Q2 (1.33, 1.19–1.54)	−0.18	0.30	−0.60	.551	0.84 (0.46–1.51)
Q3 (1.74, 1.55–2.03)	0.66	0.33	1.97	.049	1.93 (1.01–3.70)
Q4 (2.42, 2.05–13.01)	0.06	0.30	0.20	.844	1.06 (0.58–1.93)

CI = confidence interval, d = days, OR = odds ratio, PCR = polymerase chain reaction, SE = standard error, β = regression coefficient.

Bold text indicates statistical significance with *P* < .05.

**Table 3:** Multivariate logistic regression analysis results of PCR-fluorescent probe.

Variables	*β*	SE	*Waldχ*2	*P*	OR (95% CI)
Number of samples					
Single					1.00 (Reference)
Double	1.11	0.30	3.70	< .001	3.03 (1.69–5.44)
obtaining samples through surgery					
No					1.00 (Reference)
Yes	1.10	0.37	2.96	.003	3.01 (1.45–6.26)
Age					
Q1 (22, 4–25)					1.00 (Reference)
Q2 (29, 26–31)	0.95	0.37	2.55	.011	2.58 (1.24–5.33)
Q3 (36, 32–45)	0.55	0.35	1.56	.119	1.73 (0.87–3.44)
Q4 (56, 46–83)	−0.51	0.34	−1.51	.132	0.60 (0.31–1.17)
Total T lymphocytes CD3+					
Q1 (673.5, 238–834)					1.00 (Reference)
Q2 (971, 841–1100)	0.99	0.49	2.00	.045	2.69 (1.02–7.07)
Q3 (1247, 1101–1456)	0.21	0.62	0.33	.739	1.23 (0.36–4.19)
Q4 (1743, 1457–4058)	0.43	0.80	0.54	.590	1.54 (0.32–7.38)
T helper CD3 + CD4+					
Q1 (371, 112–468)					1.00 (Reference)
Q2 (558.5, 469–637)	−1.46	0.52	−2.81	.005	0.23 (0.08–0.64)
Q3 (722, 640–838)	−1.36	0.69	−1.97	.049	0.26 (0.07–0.99)
Q4 (1015, 839–1799)	−1.75	0.87	−2.00	.045	0.17 (0.03–0.96)
T helper/ T suppressor cells					
Q1 (0.93, 0.35–1.18)					1.00 (Reference)
Q2 (1.33,1.19–1.54)	0.01	0.36	0.03	.978	1.01 (0.50–2.06)
Q3 (1.74,1.55–2.03)	1.11	0.42	2.63	.008	3.03 (1.33–6.92)
Q4 (2.42,2.05–13.01)	0.90	0.46	1.95	.051	2.47 (0.99–6.14)

CI = confidence interval, OR = odds ratio, PCR = polymerase chain reaction, SE = standard error.

Bold text indicates statistical significance with *P* < .05.

### 3.3. Correlation analysis of age, Total T lymphocytes CD3+, T helper lymphocytes, and T helper/T suppressor lymphocyte ratio with PCR-fluorescent probe test results

The logistic regression analysis model results showed that, with the lowest quartile as a reference and after adjusting for confounding factors, the second quartile group for age (odds ratio [OR] = 2.95, 95% confidence interval [CI]: 1.41–6.19) and the third quartile group for T helper/T suppressor cells CD4+ CD8+ (OR = 2.75, 95% CI: 1.29–5.85) increased the risk of positive PCR-fluorescent probe test results. The third quartile group for total T lymphocytes (OR = 0.42, 95% CI: 0.21–0.87) reduced the risk of positive PCR-fluorescent probe test results. The second, third, and fourth quartile groups for T helper lymphocytes CD3 + CD4+ all reduced the risk of positive PCR-fluorescent probe test results, with a decreasing trend in risk (*P*_trend_ < .05). As shown in Table 4.

**Table 4:** Logistic regression analysis of age, total T lymphocytes CD3+, T helper lymphocytes, and T helper/T suppressor lymphocyte ratio with PCR-Fluorescent probe test results in patients with lymph node tuberculosis.

Variables	Model l		Model 2		Model 3	
	OR (95% CI)	*P*	OR (95% CI)	*P*	OR (95% CI)	*P*
Age M Value						
Q1 (22)	1.00 (Reference)		1.00 (Reference)		1.00 (Reference)	
Q2 (29)	2.37 (1.20–4.67)	.013	2.37 (1.19–4.72)	.014	2.95 (1.41–6.19)	.004
Q3 (36)	1.86 (1.00–3.45)	.050	1.90 (1.01–3.59)	.048	1.87 (0.94–3.75)	.075
Q4 (56)	0.73 (0.41–1.29)	.275	0.74 (0.40–1.38)	.344	0.99 (0.45–2.16)	.973
*P*_trend_		.026		.037		.508
Total T lymphocytes CD3+ M value						
Q1 (673.5)	1.00 (Reference)		1.00 (Reference)		1.00 (Reference)	
Q2 (971)	1.14 (0.58–2.22)	.706	1.15 (0.58–2.27)	.687	1.19 (0.56–2.53)	.653
Q3 (1247)	0.50 (0.27–0.92)	.026	0.46 (0.25–0.86)	.016	0.42 (0.21–0.87)	.019
Q4 (1743)	0.65 (0.35–1.21)	.170	0.59 (0.31–1.13)	.112	0.46 (0.21–1.00)	.051
*P*_trend_		.057		.029		.013
T helper CD3 + CD4+ M value						
Q1 (371)	1.00 (Reference)		1.00 (Reference)		1.00 (Reference)	
Q2 (558.5)	0.57 (0.29–1.10)	.093	0.53 (0.27–1.04)	.067	0.34 (0.16–0.76)	.009
Q3 (722)	0.50 (0.26–0.96)	.038	0.47 (0.24–0.91)	.025	0.33 (0.15–0.73)	.006
Q4 (1015)	0.46 (0.24–0.88)	.018	0.42 (0.22–0.83)	.012	0.23 (0.10–0.54)	< .001
*P*_trend_		.025		.018		.003
T helper/T suppressor cells CD4 + CD8+ M value						
Q1 (0.93)	1.00 (Reference)		1.00 (Reference)		1.00 (Reference)	
Q2 (1.33)	0.84 (0.46–1.51)	.551	0.88 (0.48–1.60)	.674	0.82 (0.42–1.58)	.546
Q3 (1.74)	1.93 (1.01–3.70)	.049	2.11 (1.08–4.12)	.028	2.75 (1.29–5.85)	.008
Q4 (2.42)	1.06 (0.58–1.93)	.844	1.13 (0.61–2.08)	.703	1.54 (0.75–3.17)	.245
*P*_trend_		.455		.368		.087

Model 1: Unadjusted; Model 2: Total T lymphocytes, T helper lymphocytes, T helper/T suppressor cells adjusted: sex, age, BMI, smoking, drinking; Age adjusted: gender, BMI, smoking, drinking; Model 3: Total T lymphocytes, T helper lymphocytes, T-helper/T suppressor cells adjusted: gender, age, BMI, obtaining samples through surgery, antituberculosis treatment regimen, course of disease, treatmite, initial treatment, smoking, drinking, anemia, hypoproteinemia, Tuberculosis of other organs, diabetes mellitus, hypertension, hepatobiliary diseases, number of samples, T helper T suppressor CD4CD8, adenosine deaminase; Age-adjusted: gender, BMI, obtaining samples through surgery, antituberculous regimen, course of disease, treatmite, initial treatment, smoking, drinking, anemia, hypoproteinemia, Tuberculosis of other organs, diabetes mellitus, hypertension, hepatobiliary diseases, number of samples, T helper T suppressor CD4+ CD8+, adenosine deaminase.

BMI = body mass index, CD = cluster of differentiation, CD3+ = total T lymphocytes, CD3+ CD4+ = T helper lymphocytes, CD4+ CD8+ = T helper/T suppressor cells, CI = confidence interval, OR = odds ratio, PCR = polymerase chain reaction.

### 3.4. Dose-response relationship between age, T lymphocyte subsets, and PCR-fluorescent probe test results

**Figure 2.**
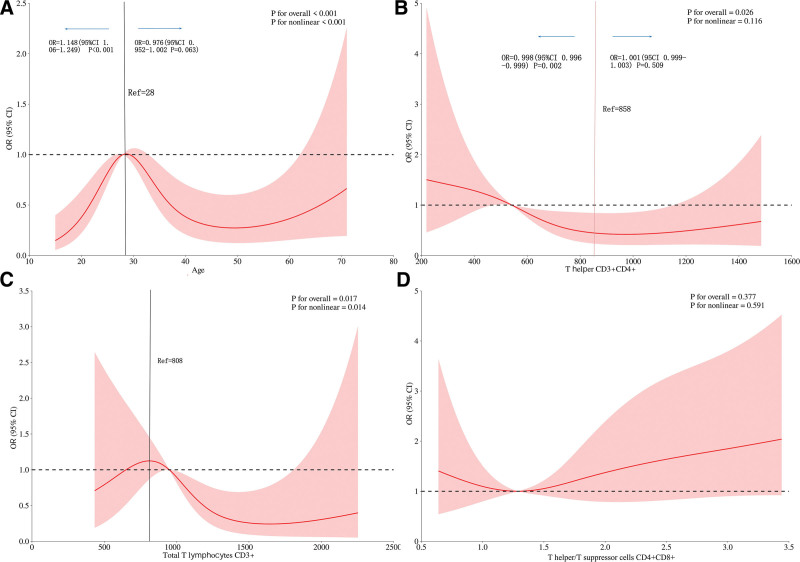
The nonlinear relationship between age, T helper CD3+ CD4+,Total T lymphocytes CD3+,T helper/T suppressor cells CD4+ CD8+ and PCR-fluorescent probe results, as fitted by logistic regression models with multivariate adjusted RCS analyses. The solid line represents the predicted values, and the shading indicates the 95% CI. It was adjusted for gender, BMI, obtaining samples through surgery, antituberculous regimen, course of disease, treatmite, initial treatment, smoking, drinking, anemia, hypoproteinemia, Tuberculosis of other organs, diabetes mellitus, hypertension, hepatobiliary diseases, number of samples, T helper T suppressor CD4+ CD8+, adenosine deaminase. BMI = body mass index, CD = cluster of differentiation, CI = confidence interval, OR = odds ratio, PCR = polymerase chain reaction, RCS = restricted cubic spline, Ref = reference value.

After adjusting for relevant confounding factors, there is a nonlinear dose-response relationship between age (*P*_total trend_ < .001, *P*_nonlinear_ < .001), total T lymphocytes CD3+ (*P*_overall trend_ = .017, *P*_nonlinear_ = .014) and the risk of positive PCR-fluorescent probe test results. There is a linear dose-response relationship between T helper lymphocytes CD3+ CD4+ (*P*_overall trend_ = .026, *P*_nonlinear_ = .116) and the risk of positive PCR-fluorescent probe test results. There is no dose-response relationship between T helper/T suppressor cells CD4+ CD8+ (*P*_overall trend_ > .05, *P*_nonlinear_ > .05) and the risk of positive PCR-fluorescent probe test results, as shown in Figure 2. Some subgroup analyses had relatively wide 95% CIs, indicating limited precision. As these are exploratory findings, they should be interpreted cautiously and not overinterpreted.

## 4. Discussion

Lymph node aspirate smear microscopy for acid-fast bacilli and *Mycobacterium* culture are conventional diagnostic methods for LNTB, but they suffer from low sensitivity and long turnaround times. Molecular assays targeting MTB DNA exhibit substantially higher diagnostic yield. Real-time fluorescent quantitative PCR with Taqman probes enables rapid identification and differentiation of MTB from nontuberculous mycobacteria within 1 working day.^[11]^ In this study, the PCR-fluorescent probe assay achieved a positivity rate of 71%, markedly higher than that of traditional bacteriological methods (10%–32%).^[12]^ Melting curve analysis also identified drug resistance in 94% (55/58) of positive cases, facilitating prompt clinical decision‑making. However, PCR positivity remains variable and is affected by sample quality and quantity. Insufficient or inappropriate sampling often leads to false-negative results and missed opportunities for drug resistance testing. This study aimed to identify key factors influencing PCR-fluorescent probe positivity and to optimize clinical sampling strategies for LNTB.

Extrapulmonary TB, unlike pulmonary TB, is categorized as a paucibacillary disease,^[13]^ meaning it has fewer bacterial counts in samples. The positivity rates for bacteriological cultures are lower due to factors such as the duration of antitubercular treatment and the patient’s history with TB. However, molecular biology techniques like PCR with fluorescent probes and GeneXpert MTB/rifampicin are not affected by these factors.^[14]^ These methods can detect residual DNA fragments in purulent or tissue samples even after bacterial cell death and lysis, thereby helping to determine drug resistance in patients. Nevertheless, some patients diagnosed with LNTB still test negative through molecular biology tests on tissue and purulent samples. Our study found that employing a strategy involving surgical sampling and the submission of multiple samples (e.g., granulation tissue + granulation tissue, granulation tissue + purulent, or purulent + purulent) can nearly double the positivity rate. This method is determined by the variability of the patient’s lesions. However, surgical sampling should be performed cautiously and only when definite surgical indications exist, balancing diagnostic yield, procedural risks, patient safety, and medical resource utilization; it is not suitable for all patients. When clinically indicated, surgical sampling and increasing specimen quantity may improve the positivity rate of MTB detection, while reducing the risk of poor wound healing and sinus formation associated with puncture procedures.

LNTB is more commonly seen in children, young adults, and females,^[6,15]^ although it can also occur in the elderly and those with weakened physical health. With the widespread implementation of Bacillus Calmette-Guérin vaccination in children, there has been a decline in MTB infection rates among this demographic, shifting the peak age of LNTB to between 20 and 45 years old.^[16,17]^ Gouda^[18]^ proposed that PCR-fluorescent probe methods are not influenced by age or site of infection. However, our study, utilizing a RCS model, found a nonlinear dose-response relationship between age and PCR-fluorescent probe results, with an inflection point at 28 years old. The increasing risk of PCR positivity before age 28 is consistent with the active immune response and relatively higher mycobacterial burden in young adults, who constitute the main epidemiological peak of LNTB. After 28 years of age, cellular immune function gradually stabilizes and mycobacterial clearance is enhanced, leading to a progressive reduction in tissue bacterial nucleic acid load and lower PCR positive risk. The risk of positive PCR-fluorescent probe results shows an increasing trend for those under 28 years and a decreasing trend for those over 28 years. This aligns with the common age range for LNTB, indicating that individuals aged 26 to 31 years are more likely to have positive test results. These age-related changes reflect the combined effects of immune maturation, mycobacterial burden, and disease activity, rather than an independent effect of age itself. In other age groups, increasing the volume of samples submitted for testing can improve the likelihood of obtaining positive results and further conducting drug resistance screening. However, the specific mechanisms behind this require further investigation.The immune response induced by MTB is primarily cell-mediated immunity led by T cells. Studies have confirmed that the absolute count of CD3+ T cells in patients over 60 years old may be significantly lower than in those under 60.^[19]^ Factors such as anemia and hypoproteinemia can also lead to a decrease in the total CD3+ T lymphocyte count.^[20]^ After adjusting for these confounding factors, our study found a nonlinear dose-response relationship between total CD3+ T lymphocytes and PCR-fluorescent probe results, with an inflection point at 808 cells/μL. Below 808 cells/μL, cellular immunity is suppressed; as CD3 + T-cell levels rise, antituberculosis immunity gradually strengthens, leading to increased mycobacterial lysis and nucleic acid release, thus elevating the PCR positive rate. When immunity is further enhanced beyond 808 cells/μL, efficient mycobacterial and nucleic acid clearance results in a gradual decline in PCR positivity. Notably, these inflection points represent exploratory findings derived from retrospective analysis and require prospective multicenter validation prior to clinical application.CD4+ T cells play a central role in the cellular immune response and have been confirmed to be crucial in the immune response against MTB infection.^[21]^ CD4+ T cells participate in the clearance of MTB.^[22,23]^ Li et al (2020) further verified that CD4+ T-cell count is negatively correlated with mycobacterial load and lesion severity in active TB, providing direct evidence supporting our findings. Our study shows a linear dose-response relationship between helper T lymphocyte counts (CD3+ CD4+) and the risk of positive PCR-fluorescent probe results, with an inflection point at 858 cells/μL. For helper T lymphocyte counts below 858 cells/μL, the risk of positive PCR-fluorescent probe results gradually decreases; above 858 cells/μL, the change in risk is not significant. This linear pattern and threshold effect demonstrate that stronger CD4+-mediated immune clearance reduces mycobacterial nucleic acid content in lesions, thereby lowering PCR positivity; once the protective immune threshold is reached, further increases in CD4+ cells produce no additional significant effect. This suggests that as more CD4+ T cells participate in the clearance of MTB, the probability of detecting a positive result with PCR-fluorescent probe decreases, but after reaching a certain threshold, no significant changes occur. Therefore, for patients whose reference values exceed the threshold of this test, if the surgical indications are met, we can prioritize obtaining samples through surgery, increase the amount of postoperative samples for testing, and reduce the adverse consequences caused by puncture.

## 5. Limitations

This study has certain limitations: This was a single-center, retrospective observational study that only enrolled patients with LNTB. Since tissue characteristics, bacterial load, and anatomical features differ substantially across other types of extrapulmonary TB, the present findings cannot be generalized to other extrapulmonary TB populations or clinical scenarios; Patient selection was restricted to a single center in Xi’an, and institutional practice patterns (including specimen collection protocols, surgical indication criteria, and laboratory detection procedures) may also influence the detected results. Therefore, the generalizability of our conclusions to other regions and medical settings is limited; The retrospective design precludes us from drawing causal inferences between age, T lymphocyte subsets, and PCR-fluorescent probe test outcomes. Future large‑scale, multicenter, prospective cohort studies are warranted to verify and validate these preliminary findings.

## 6. Conclusion

This study demonstrates that surgical sampling and increasing the number of specimens significantly improve the positivity rate of PCR‑fluorescent probe testing in patients with LNTB. Of note, surgical sampling should be performed only when clinically indicated, balancing diagnostic yield, procedural risks, and medical resource utilization. Age, total CD3+ T lymphocytes, and helper T lymphocytes (CD3+ CD4+) exhibit linear or nonlinear dose‑response relationships with PCR positivity. These findings suggest that age and CD3+ CD4+ T lymphocytes may serve as potential indicators for predicting PCR positivity.

## Acknowledgments

We thank the reviewer for the constructive feedback, which helped us further improve the clarity and originality of the manuscript.

## Author contributions

**Conceptualization:** Qian Li, Chao Ding.

**Data curation:** Qian Li, Chao Ding, Leipeng Ren.

**Formal analysis:** Qian Li, Chao Ding.

**Funding acquisition:** Chao Ding.

**Investigation:** Peijia Luo.

**Methodology:** Peijia Luo.

**Project administration:** Peijia Luo, Dangze Sun.

**Resources:** Dangze Sun.

**Software:** Dangze Sun.

**Supervision:** Leipeng Ren.

**Validation:** Leipeng Ren.

**Visualization:** Leipeng Ren.

**Writing – original draft:** Qian Li.

**Writing – review & editing:** Leipeng Ren.
